# Variable Fitness Impact of HIV-1 Escape Mutations to Cytotoxic T Lymphocyte (CTL) Response

**DOI:** 10.1371/journal.ppat.1000365

**Published:** 2009-04-03

**Authors:** Ryan M. Troyer, John McNevin, Yi Liu, Shao Chong Zhang, Randall W. Krizan, Awet Abraha, Denis M. Tebit, Hong Zhao, Santiago Avila, Michael A. Lobritz, M. Juliana McElrath, Sylvie Le Gall, James I. Mullins, Eric J. Arts

**Affiliations:** 1 Division of Infectious Diseases, Department of Medicine, Case Western Reserve University, Cleveland, Ohio, United States of America; 2 Vaccine and Infectious Disease Institute, Clinical Research Division, Fred Hutchinson Cancer Research Center, Seattle, Washington, United States of America; 3 Department of Microbiology, University of Washington, Seattle, Washington, United States of America; 4 Partners AIDS Research Center, Massachusetts General Hospital, Harvard Medical School, Boston, Massachusetts, United States of America; 5 National Institute of Respiratory Diseases, Center for Research in Infectious Diseases, Mexico City, Mexico; 6 Department of Molecular and Microbiology, Case Western Reserve University, Cleveland, Ohio, United States of America; Nationwide Children's Hospital, United States of America

## Abstract

Human lymphocyte antigen (HLA)-restricted CD8^+^ cytotoxic T lymphocytes (CTL) target and kill HIV-infected cells expressing cognate viral epitopes. This response selects for escape mutations within CTL epitopes that can diminish viral replication fitness. Here, we assess the fitness impact of escape mutations emerging in seven CTL epitopes in the gp120 Env and p24 Gag coding regions of an individual followed longitudinally from the time of acute HIV-1 infection, as well as some of these same epitopes recognized in other HIV-1-infected individuals. Nine dominant mutations appeared in five gp120 epitopes within the first year of infection, whereas all four mutations found in two p24 epitopes emerged after nearly two years of infection. These mutations were introduced individually into the autologous gene found in acute infection and then placed into a full-length, infectious viral genome. When competed against virus expressing the parental protein, fitness loss was observed with only one of the nine gp120 mutations, whereas four had no effect and three conferred a slight increase in fitness. In contrast, mutations conferring CTL escape in the p24 epitopes significantly decreased viral fitness. One particular escape mutation within a p24 epitope was associated with reduced peptide recognition and high viral fitness costs but was replaced by a fitness-neutral mutation. This mutation appeared to alter epitope processing concomitant with a reduced CTL response. In conclusion, CTL escape mutations in HIV-1 Gag p24 were associated with significant fitness costs, whereas most escape mutations in the Env gene were fitness neutral, suggesting a balance between immunologic escape and replicative fitness costs.

## Author Summary

Upon infection with a pathogen, the host mounts an immune response of specific killing by recognizing infected cells presenting the foreign entity via host cell surface proteins (aka MHC) encoded by specific HLA genes. The pathogen's proteins are chopped up into peptides (short protein sequences) by cellular enzymes. Some of these peptides are bound to MHC class I proteins and then presented on the cell surface, signifying a pathogen intrusion to the killer T cells (CTLs). In order to survive, HIV-1 evolves to mutate the specific peptides (aka epitopes) and escape recognition by MHC I and CTLs. These “forced” or selected mutations are thought to come at a cost, e.g., result in slower pathogen growth/replication. Here, we show that the escape mutations in HIV-1 genes showing slow evolution result in dramatic losses in HIV-1 replication (or fitness). In contrast, CTL escape mutations in HIV-1 genes with rapid evolution do not have a negative impact on pathogen fitness. These findings could suggest that HIV-1 (and possibly other rapidly evolving pathogens) could avoid the host's immune system with minimal detriment to its own survival and growth within the host.

## Introduction

It is well established that the CTL response plays a major role in control of both acute and chronic HIV-1 infection (reviewed in [1]). Thus, numerous vaccine strategies are being evaluated to elicit broad and potent CTL responses against HIV proteins [2]. However, a major obstacle is CTL-mediated selection leading to viral escape, which occurs frequently in acute/early [3]–[8] and chronic [9]–[11] infection. Recent studies have shown that CTL selection is a major force driving viral evolution within individual patients [12]–[14] and at the population level [15]–[18]. For some epitopes, escape mutations are often slow to emerge, or even absent despite strong specific CTL responses [13],[19],[20]. This rate of CTL escape during infection may be influenced by mutational pathways required for escape (e.g., requirements for transversion versus transition and single versus multiple nucleotide mutations), the killing efficiency (or “strength”) of CTL responses [1] as well as stochastic processes [21]. However, another often cited factor for the slow appearance of CTL escape mutation may be the “cost” on replicative fitness of the infecting strain [22].

Several lines of experimental evidence have inferred fitness costs. First, specific HIV-1 and SIV escape variants can be maintained in viral populations after transmission to a host sharing the restricting HLA allele, and revert to the consensus, epitopic form when transmitted to an individual not sharing the donor restricting HLA allele [13], [23]–[25]. Thus, in the absence of specific CTL responses, viruses with consensus amino acids are likely to have higher replicative fitness. Second, some escape mutations can be compensated by extra-epitopic mutations also under selection, implying a fitness cost for the escape mutation [26]–[29]. Lastly, introduction of CTL escape mutations into HIV-1 and SIV epitopes has been shown to reduce replication kinetics [26], [30]–[34]. Martinez-Picado *et al.* have provided evidence that a specific T242N mutation in the TW10 Gag p24 epitope can reduce fitness when introduced into the autologous (patient-derived) HIV-1 sequence [32]. However, the collective assumption that CTL escape mutations result in fitness loss is mainly derived from analyses of discrete mutations in Gag epitopes of heterologous, laboratory HIV-1 strains.

We recently reported a comprehensive analysis of viral evolution and CTL recognition in one subject over the first three years of infection with clade B HIV-1 [13]. CTL epitopes were characterized by IFN-γ ELISPOT using consensus and autologous peptides spanning the entire HIV-1 proteome during first three years of infection [13]. In the current study, we assessed the fitness cost of all 13 epitopic mutations observed in Gag p24 and Env gp120. Multiple selective factors may shape viral evolution especially in surface exposed envelope glycoproteins but in this study, we examined the phenotypic effects of mutations that emerge within or around CTL epitopes, and focused on those conferring CTL escape. Replicative fitness was measured by competing a virus with a CTL escape mutation in the autologous virus backbone against its “parental” clone. In general, introduction of epitopic escape mutations into gp120 Env had either no effect or enhanced *ex vivo* fitness, while a significant fitness loss was evident with escape mutations into the autologous p24 Gag sequence.

## Results

### Schema for producing autologous HIV-1 chimeras to test fitness costs

**Figure 1:**
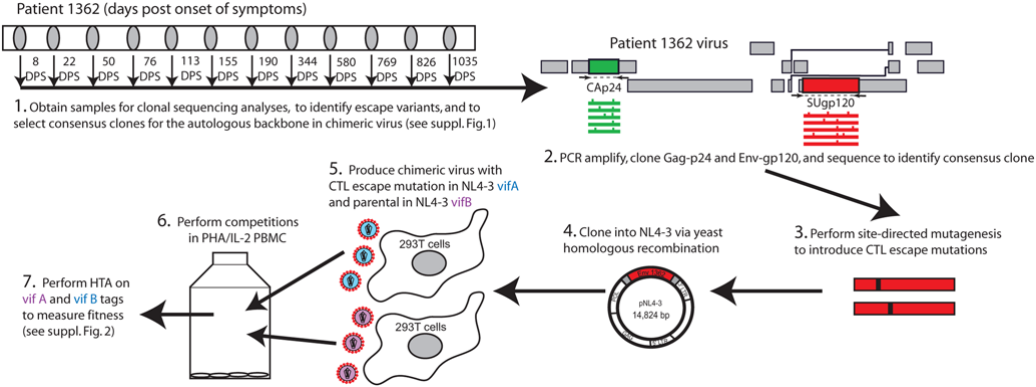
Experimental Strategy. In part 1, the days post onset of symptoms (DPS) viral genetic data was obtained from plasma in our previous study. In addition, sequences were obtained from PBMC DNA from days 34 and 298 for the present study (part 2). The remaining parts of the Figure outline the methods used to generate isogenic viruses and perform *ex vivo* competition assays in PBMC. Additional details are provided in Figure S1 and S2.

**Figure 2:**
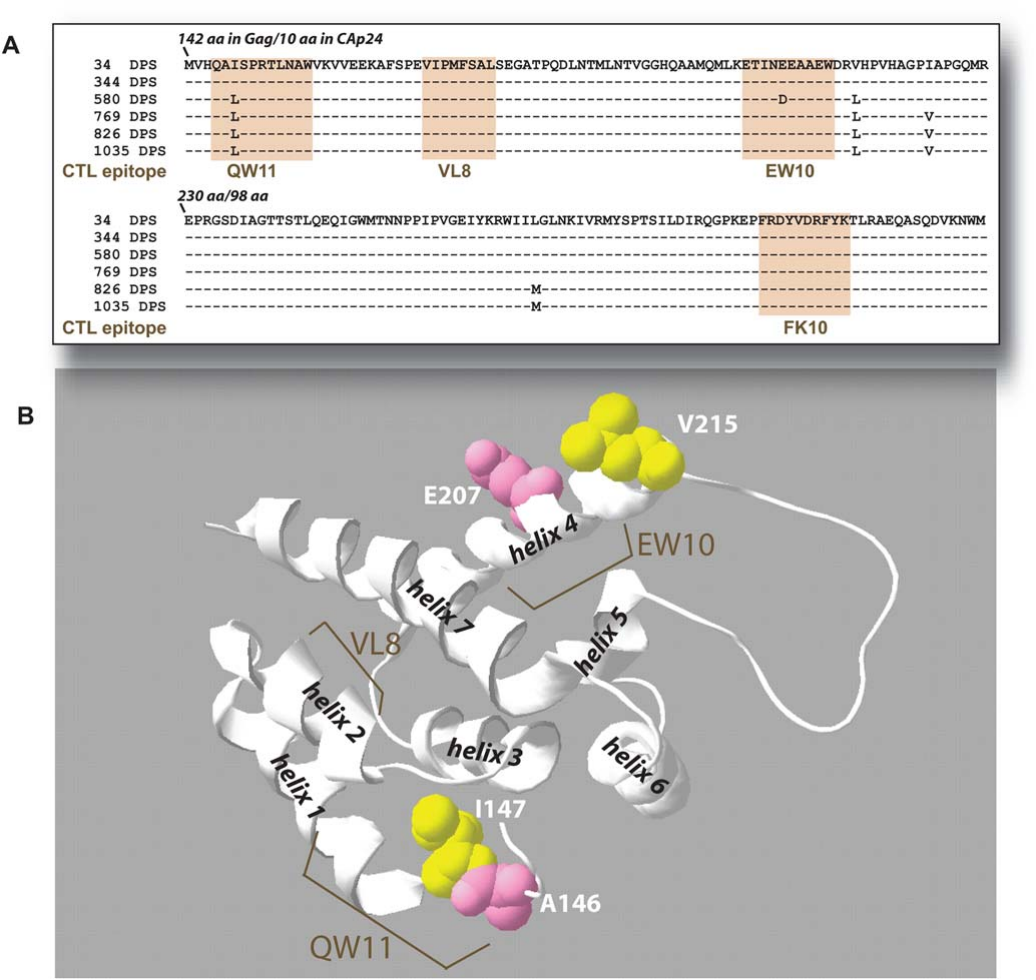
Mapping of the CTL escape and secondary mutations of PIC1362 onto the primary amino acid sequence and X-ray crystal structures of the N-terminus of p24 Gag. CTL epitopes are mapped to the consensus amino acid sequences of p24 in panel (A) (shaded regions). Amino acid positions are indicated using HXB2 amino acid numbering. Sites of mutations are depicted as space filled amino acids (those within the epitopic form are shown) in X-ray crystallography-derived ribbon structure models of the N terminus p24 [36] (B). The “yellow” residues signify sites at which no impact on replication fitness was detected and “pink” – decreased fitness (see Figure 4).

**Figure 3:**
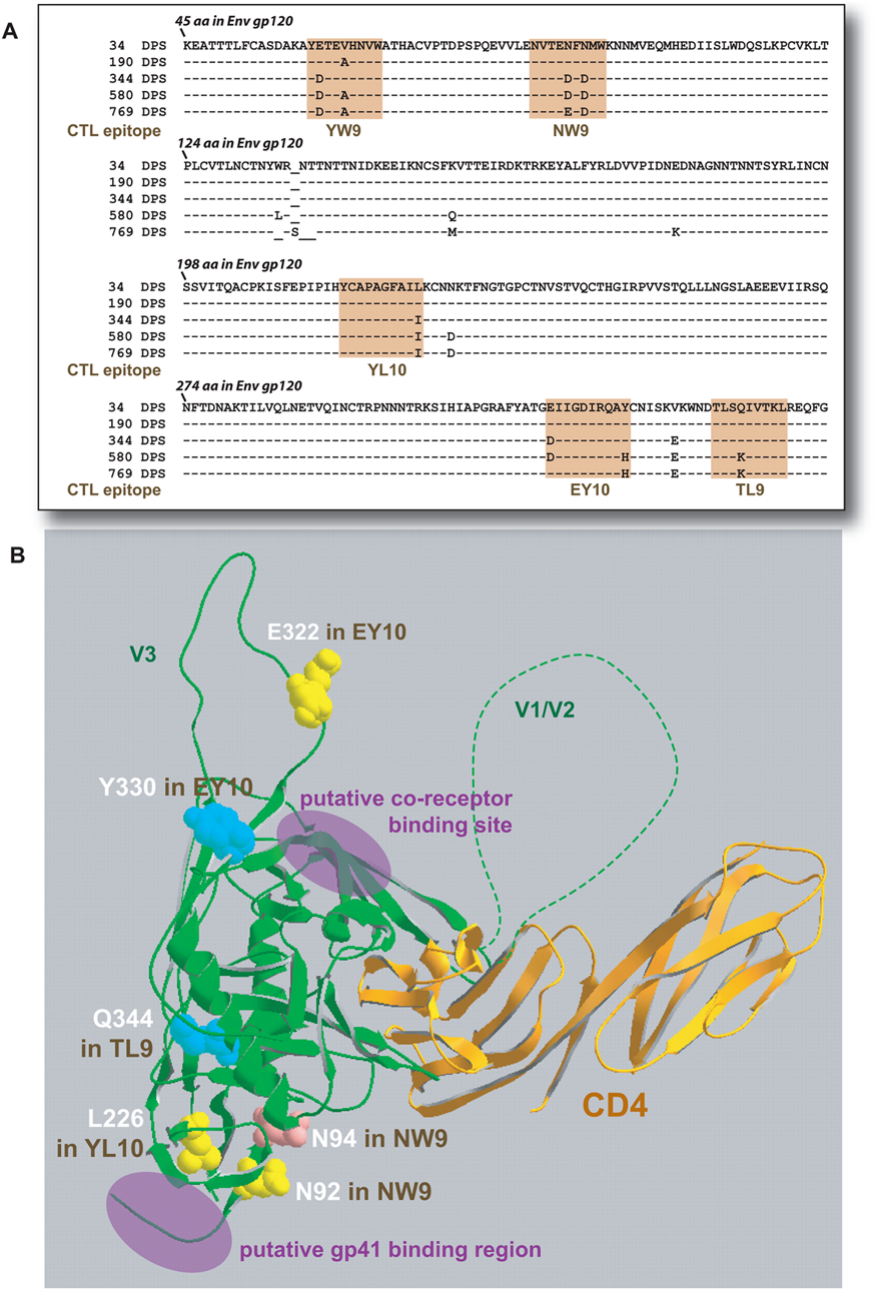
Mapping of the CTL escape and secondary mutations of PIC1362 onto the primary amino acid sequence and X-ray crystal structures of the N-terminus of gp120 Env core. CTL epitopes are mapped to the consensus amino acid sequences of gp120 in panels (A) (shaded regions). Amino acid positions are indicated using HXB2 amino acid numbering. Sites of mutations are depicted as space filled amino acids (those within the epitopic form are shown) in X-ray crystallography-derived ribbon structure models of the gp120 core complexed to CD4 and an anti-gp120 antibody [37] (D). The “yellow” residues signify sites at which no impact on replication fitness was detected, “pink” – decreased fitness, and “blue” - increased fitness (see Figure 6).

Patient 1362 was recruited into the Seattle Acute Primary Infection Cohort (PIC) 8 days post (onset) development of symptoms (DPS) of primary HIV-1 infection (Figure 1). Twenty-five CTL epitopes were identified over 12 time points during the first 3 years of infection [13], and one-third of the amino acid sites identified as undergoing selection were associated with mutational escape from CTL. A total of 10 epitopes were detected in the Gag capsid protein p24 and in the Env glycoprotein gp120 (Figure 2). One of six gp120 epitopes was found at the N-terminus [35] and was not part of the cloned gp120 cassette used in this study. Escape mutations were detected in two of the four Gag epitopes (at 580 and 769 DPS) (Figure 2A) and in all five gp120 epitopes by 113 to 580 DPS (Figure 2B). Position of these mutations and of the CTL epitopes in the HIV-1 p24 and gp120 coding regions are illustrated in Figure 2A and C, respectively. Sites of predicted amino acid substitutions within or near these epitopes were also mapped to the p24 [36] and gp120 crystal structures [37] (Figures 2B and 3B, respectively). For the current analyses, p24 and gp120 coding regions were PCR-amplified from patient 1362 PBMC and then cloned into an HIV-1 NL4-3 background genome by yeast homologous recombination (Figure 1, Figure S1 and Protocol S1 online) [38]. We have previously reported on the cloning of two CTL escape mutations in one of the p24 epitopes (EW10) [35]. However, competition and fitness analyses were still repeated for these studies.

We first selected p24 and gp120 plasmid clones matching the PIC1362 consensus HIV-1 sequence at 8 DPS and prior to the appearance of CTL escape mutations. These clones served as the autologous genetic backbone for introduction of CTL escape mutations by site-directed mutagenesis. Chimeric NL4-3 harboring these autologous p24 and gp120 sequences were shown to be replication competent (see below), and mutations were introduced into one epitope at a time for study. Epitopic mutations within seven p24 and gp120 epitopes, corresponding to a total of 13 CTL escape and secondary mutations appearing over the first three years of infection, were introduced into the PIC1362 HIV-1 sequence alone or in combination to produce 6 NL4-3/p24 and 13 NL4-3/gp120 mutant chimeric viruses for fitness analyses (see below). Full-length chimeric viruses were constructed, produced, and quantified as outlined in Figure 1 (details provided in Figure S1 online).

To measure replicative fitness, equal infectious units of viruses harboring CTL escape mutations (in the vifA backbone) were competed against the parental virus (vifB) in PBMC cultures. In these dual infection/competition assays, the production of the vifA virus, harboring six synonymous substitutions, can be differentiated from the vifB virus using a quantitative heteroduplex tracking assay (assay details and examples of results provided in Figure S2 online) [39],[40]. To ensure that the vif substitutions did not impact fitness, competitions were performed using NL4-3_vifA_ and NL4-3_vifB_ virus harboring the same PIC1362 p24 (or gp120) sequence. These viruses had equal fitness, confirming [39],[40] that the vif synonymous substitutions did not impair replication (grey bars in Fig. 3 and see below) (p>0.1, n = 8).

### Effects of CTL escape mutations in the HIV-1 capsid protein on replication fitness

Of the four CTL epitopes that mapped to p24, escape mutations emerged in the two HLA-A25-restricted QW11 and EW10 epitopes over the first 3 years of infection (Figure 2A), but not in VL8 and FK10 epitopes (Figure 2A), despite strong CTL responses during the 1035 days of follow-up [6] (and data not shown). Failure to detect escape mutations in these epitopes may be related to the defective phenotypes conferred by mutations [41] as discussed below (Figure 2B).

**Figure 4:**
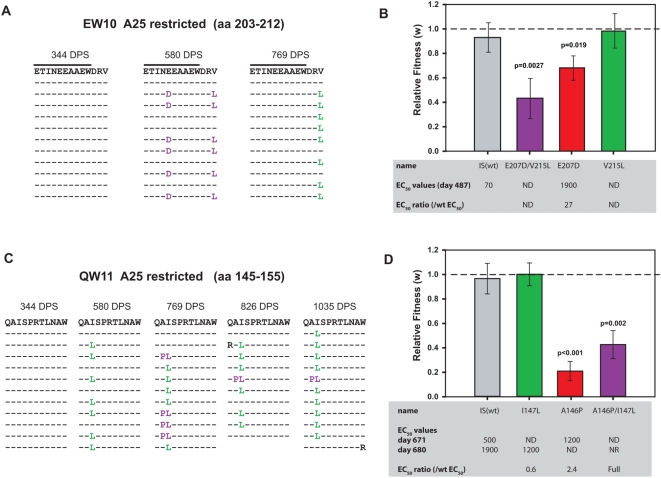
Ex vivo fitness, CTL escape phenotype and sequence evolution of *gag* p24 CTL escape mutants. The predicted amino acid sequence derived from 11–12 clones at different DPS is presented for epitopes EW10 (A) and QW11 (C). The effect of each mutation, or combination of mutations, on the CTL responses to the epitopic peptide in PIC1362 was assessed by ELISpot (grey box in panels (B) and (D)). The fold escape conferred by each mutation is displayed here as the ratio of effective peptide concentration (EC_50_) required to elicit 50% of maximal ELISpot signal relative to the pre-escape peptide [54]. EC_50_ values>10 were considered to indicate escape. The standard IFN-γ Elispot assay was performed with the defined optimal epitopic peptide at the following concentrations: 0.64, 3.2, 16, 80, 400, 2,000, and 10,000 ng/ml. The effective peptide concentration that elicited 50% of the maximum T-cell response, defined as the EC50, was determined with the Sigmoidal Fit tool in the software. ELISpot assays not done (ND) with some peptides. NR (no response) refers to a lack of response to a mutant peptide by ELISpot. A positive response was defined as twice the negative control and >50 Spot Forming Cells (SFC)/10^6^ cells. In the case where the wild type peptide was recognized and mutant peptide did not induce a response, full escape (“Full”) was given as the value for the EC_50_ ratio. Mutations that conferred significant CTL escape are displayed in red columns in (B) and (D). Mutations that did not confer significant escape are shown in green type in (A) and (C) and in green bars in (B) and (D). Combinations of these mutations are shown in purple. CTL escape and associated mutations in the p24 coding sequence were introduced individually and in combination into chimeric NL4-3 viruses bearing the consensus initial/infecting strain from 8DPS (Figure 1 and Figure S1). The effect of these mutations was determined by direct competition with isogenic virus bearing the wild-type amino acid. The control involved competitions between 8DPS-p24 virus competed against a virus of identical amino acid sequence (except for synonymous mutations in *vif* to differentiate the viruses). These viruses were equally represented following dual infection and thus had equal fitness (relative fitness ∼1) (gray bar in (B) and (D)). Significance of any increase or decrease in fitness conferred by a mutation(s) was evaluated using Student's t-test to compare the relative amounts of each virus. Only p values<0.05 are displayed on the graphs and the 95% confidence interval of the mean is shown for each bar. Fitness data presented on the EW10 epitope (B) have been previously published [35] but for this figure, all competitions were repeated in quadruplicate resulting in very similar results.

An E207D mutation emerged within in the EW10 epitope at 580 DPS (Figure 4A), conferring escape (ratio of EC_50_ EW10/EC_50_ E207D = 27; Figure 4B). Interestingly, the E207D mutation was accompanied by a V215L mutation, 3 amino acids C-terminal to the epitope (Figure 4A). A significant decrease in replication fitness was observed when E207D or the E207D/V215L double mutation was evaluated (Figure 4B). However, the virus containing the V215L mutation alone did not alter fitness. The E207D escape mutation was not detected at 769 DPS, whereas the V215L mutant was dominant at that time (Figure 4A). The proximity of the V215L mutation to the EW10 epitope suggests that it may have conferred CTL escape by impairing peptide processing [31], allowing the epitopic sequence to re-emerge. Impact of E207D and V215L on peptide processing is examined below.

**Figure 5:**
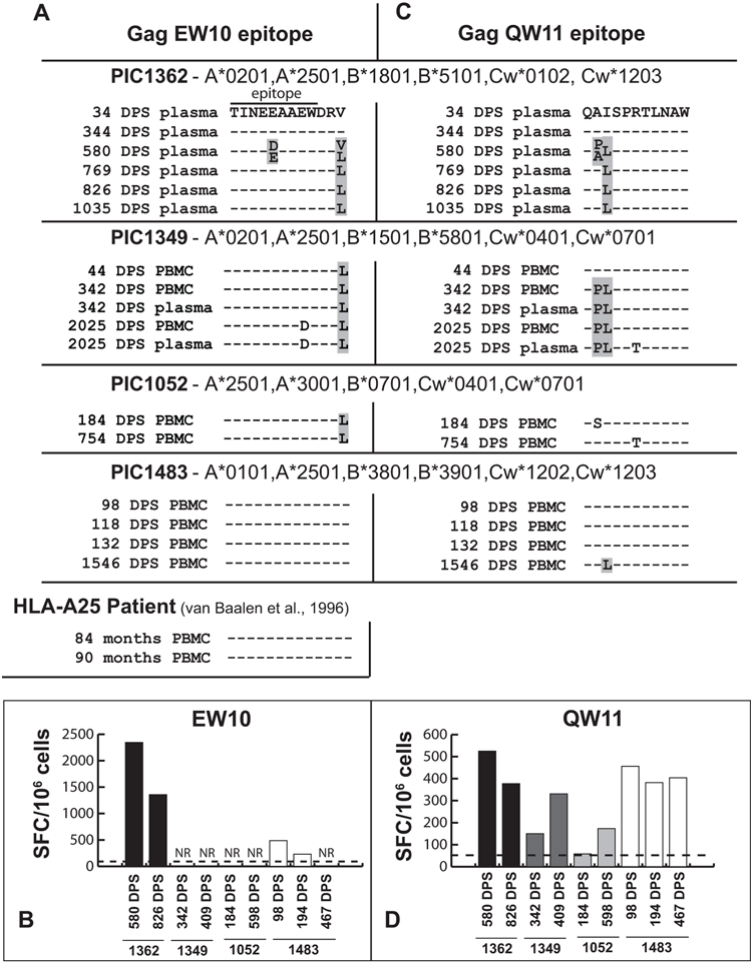
Sequence of p24 epitopes QW11 and EW10 in HLA-A25 patients with CD8+ T cell responses to these epitopes. The consensus sequence at each timepoint was determined by bulk population nucleotide sequencing of the QW11 (A) and EW10 (C) epitopes of four HLA A25-restricted patients, PIC1362, PIC1349, PIC1052, and PIC1483. The sequences of the QW11 and EW10 epitopes of PIC1362 are shown at the top of panels A and C. The EW10 sequences from the “HLA-A25” patient subject at the bottom of panel C is from a previously published study [42]. The shaded amino acids refer to CTL escape mutations. Non-shaded epitopic mutations were associated with escape but confirmatory CTL recognition assays were not performed. ELISpot assays were performed with the QW10 and EW10 peptides and PBMC from multiple time points from PIC1362, PIC1349, PIC1052, and PIC1483 (B and D). NR refers to no detectable IFN-γ ELISpot response.

To compare CTL escape patterns in EW10, we evaluated viral sequences as well as IFN-γ secreting T cell responses (as measured by ELISpot) to EW10 in each of three other PIC subjects sharing the same A25 HLA restricting allele (PIC1052, PIC1349, and PIC1483) (Figure 5A and B). No mutations were observed in the EW10 epitope of patients PIC1052 and PIC1349. However, these patients were infected with virus containing the putative processing mutation, 215L. This was not unexpected and has been noted previously [35], the leucine at position 215 is the consensus amino acid in HIV-1 subtype B (88% of sequences), whereas valine is only found in 10% of HIV-1B sequences. Lack of ELISpot responses to EW10 in PIC1052 and PIC1349 along with the presence of 215L suggests the lack of peptide presentation. In contrast, a CTL response was detected in PIC1483, who, like PIC1362, was infected with virus bearing valine at position 215. However, the PIC1362 T cell response to the EW10 peptide was 10-fold greater than that of PIC1483 T cell (Figure 5B). Reduced CTL pressure and high fitness costs of EW10 escape variants might explain for the lack of evolution within or the near vicinity of the EW10 epitope of PIC1483 (Figure 5A). In another study [42], EW10 was recognized (and not found to escape) in a subject infected with a virus harboring the p24 215V allele (Figure 5A). In summary, this data is consistent with the hypothesis that the V215L mutation interferes with the presentation of the EW10 epitope – the epitope is recognized in three subjects that had the rare valine in the C-terminal flanking sequence, but not in the subjects that had the consensus leucine at this position.

Impact of CTL escape mutations on replicative fitness was also assessed in the other recognized, HLA A25-restricted p24 epitope of PIC1362. An I147L mutation was first detected in QW11 at 580 DPS (Figure 4C) but this did not confer an obvious escape (ratio of EC_50_ QW11/EC_50_ I147L = 0.6; Figure 4D) or a loss in viral fitness (Figure 4D). However, by 769 DPS, an adjacent mutant emerged (A146P) linked to I147L, that by itself was weakly recognized by CTL (EC_50_ ratio = 2.4), while drastically reducing viral fitness (Figure 4D). The A146P/I147L QW11 peptide was not recognized in ELISpot assays (Figure 4D). Although this double mutant did restore some replicative fitness compared to I147L alone, the virus harboring these mutations were still less fit than the parental PIC1362 virus (Figure 4D). After 769 DPS, the A146P/I147L double mutants were present in a minority of sequences while the I147L mutation remained dominant (Figure 4B). The I147L mutation may therefore provide a limited degree of compensation for the fitness loss associated with the escape mutant A146P, while facilitating epitopic escape.

To address the commonality of these observations, we assessed the recognition and stability of the QW11 epitope in the same 3 additional HLA-A25 subjects from the PIC cohort. QW11 was recognized by all three individuals, but at slightly lower levels compared to that in PIC1362 (Figure 5D). PIC1349 developed the same A146P/I147L double mutation observed in PIC1362 (Figure 5C). However, unlike PIC1362, both mutations dominated in the HIV-1 population for an extended period (from 342 through at least 2025 DPS) (Figure 5C). It is important to note that in the case of PIC1349, an overlapping epitope starting at position A146 (AW10) could be recognized through the B*5801 allele [43],[44]. CTL escape from a B58 restricted response may also be related to the emergence of A146P and/or I147L. It is not surprising that same anchor residues may be mutated to confer escape to an A25 and B58-restricted response. The I147L mutation also emerged in PIC1483, although was only detected at the last day of sampling (1546 DPS). Finally, in PIC1052, an A146S mutation emerged at 184 DPS but disappeared by 754 DPS, at which time the R150T mutation was dominant (Figure 5A). Thus, the QW11 epitope was recognized in all 4 HLA-A25 subjects and was a site of viral evolution in each case. However, we did not determine the impact of these mutations on CTL recognition nor on viral fitness. It should be noted that the emergence of specific EW10 or QW11 mutations was not associated with demonstrable increases in viral load or decreases in CD4 cell counts in any of the 4 subjects (data not shown).

### Peptide processing during evolution of the EW10 p24 epitope

Population dynamics of the HIV-1 p24 gag sequences within PIC1362 has been previously characterized [35] and summarized in Figure S3A online. The EW10 peptide dominated the population over 550 DPS with a small fraction containing the extra-epitopic mutation, V215L. A strong ELISpot response to the EW10 peptide (2345 SFC/10^6^ cells) was observed prior to the emergence of the E207D mutation (Figure S3B online). While mostly linked to V215L, E207D reached peak levels at ∼50% of the HIV-1 population around 713 DPS, which corresponded to a reduction in ELISpot response (530 SFC/10^6^ cells). This depletion of PBMCs responsive to the EW10 in PIC1362 may be explained by reduced ability to recognize the E207 peptide (Figure 4B). By 826 DPS, the loss of E207D was associated with a rebound in EW10 ELISpot response (1400 SFC/10^6^ cells) (Figure S3 online). By 1035 DPS, all wild type EW10 sequence were linked to the adjacent V215L mutation and over the next year and half, the dominant V215L in the HIV-1 population corresponded to a reduction in EW10 response (303 SFC/10^6^ cells at 1329 DPS and 262 SFC/10^6^ cells at 1501 DPS). Obviously, the extra-epitopic V215L mutation cannot directly explain for the reduction in PBMCs responding to the EW10 peptide. Alternatively, V215L may alter processing of the EW10 peptide.

**Figure 6:**
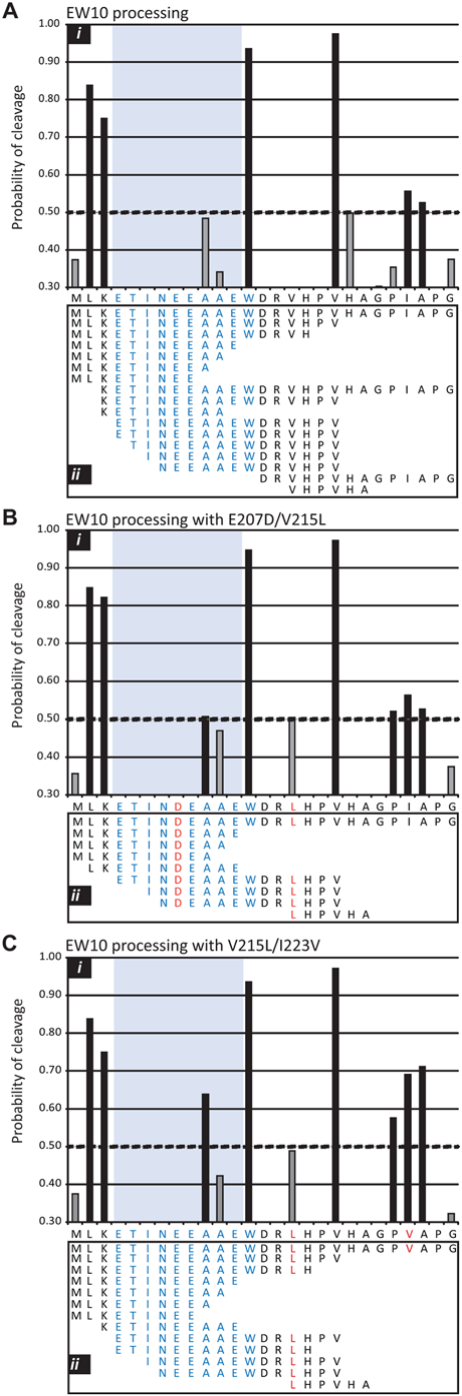
Predicted and *ex vivo* processing of peptides harboring the EW10 epitope. Peptides of 27 amino acids in length and representing positions 200 to 226 in the Env gp120 sequence were used in silico in the NetChop 3.0 C-term peptide processing algorithm (http://www.cbs.dtu.dk/services/NetChop/). C-term 3.0 network is trained with a database consisting of 1260 publicly available MHC class I ligands (using only C-terminal cleavage site of the ligands). The probability of cleavage at each amino acid (panel i) is presented for wt 27 mer peptide (A) and peptides containing the E207D/V215L (B) or V215L/I223V (C). The cleavage peptide products were predicted based on a program recommended probability of cleavage (above a 0.5 threshold, black bars). Low probabilities of cleavage (below 0.5) were presented in grey bars. The same three 27 mer peptides were also synthesized and subject to protease processing using cytosol preparation from HIV-negative PBMCs. Panel ii displays the peptides derived from the wt (A), E207D/V215L (B) or V215L/I223V (C) and identified by mass spectrometry.

To test this hypothesis, three HIV-1 peptides harboring the EW10 epitope were subject to both ex vivo cytosolic degradation and in silico proteasome processing. NetChop 3.0 was used to predict the proteosomal cleavage products generated from 27 and 40 mer peptides. These peptides used for the processing study contained the EW10 epitope and combinations of E207D, V215L, and I223V mutations found over the course of PIC1362 infection. The program C-term 3.0 network is trained with a database consisting of 1260 publicly available MHC class I ligands (using only C-terminal cleavage site of the ligands). Overall, twelve peptide products were predicted from wt 27 mer using NetChop 3.0 (Figure 6A, panel ii). The highest probabilities of cleavage (threshold set at 0.5) were observed at the C-term of K202 and W213 resulting in an intact EW10 peptide. A similar pattern of peptide cleavage was observed when the algorithm was run on longer wt peptides (data not shown). Relative proportion of products can be predicted using NetChop 3.0 (Figure 6A, panels i). The probability of cleavage at position A209 (within EW10) did increase from 0.47 with the wt 27 mer (Figure 6A, i), to 0.51 with E207D/V215L peptide (Figure 6B, i), and to a 0.64 cleavage probability with the V215L/I223V peptide (Figure 6C, i).

Whereas NetChop3.0 predicts proteasomal cleavage sites, cytosolic degradation assays take into account all peptidases (proteasomes, endopeptidases and aminopeptidases) involved in the production of epitopes and epitope precursors in the cytosol. For this assay, a wt 27 mer peptide starting at position 200 in Gag (position 66 in p24) was incubated with PBMC cytosolic extracts of an HIV-negative donor for 10 and 60 min and then subject to analyses by mass spectrometry [45]. Majority of peptides suggest cleavage events at position 202 and 213 but with some further trimming at the ends of the EW10 peptide (Figure 6A, ii). With the E207D/V215L or V215L/I223V peptides, both predictive and ex vivo processing suggest increased cleavage within the EW10, thus destroying the epitope. Fewer peptide products were generated from ex vivo processing of the E207D/V215L peptide as compared to the V215L/I223V peptide (Figure 6B and C). Unfortunately, the relative amounts of each cleavage product could not be measured by MS. However, decreased EW10 presentation is likely due to the increased probability of cleavage in EW10 with V215L (as predicted NetChop).

Based on these predictive algorithms and supported by ex vivo processing results, enhanced cleavage in EW10 was observed in the E207D/V215L double mutant. However, E207D alone, which was rarely found in PIC1362, does not alter EW10 processing (data not shown) based on predictive algorithms. Together, this data suggests that the emergence of the extra-epitotic mutation, V215L mutation augments cleavage within the EW10 epitope of HIV-1 p24. As discussed below, it is important to note that the V215L does not abolish the presentation of the EW10 peptide, which might explain for the slow reduction and retention of some EW10-responsive PBMCs after a year and half of V215L dominance in the HIV-1 population of PIC1362 (Figure S3B online).

### Effects of CTL escape mutations in the HIV-1 envelope glycoprotein on replication fitness

**Figure 7:**
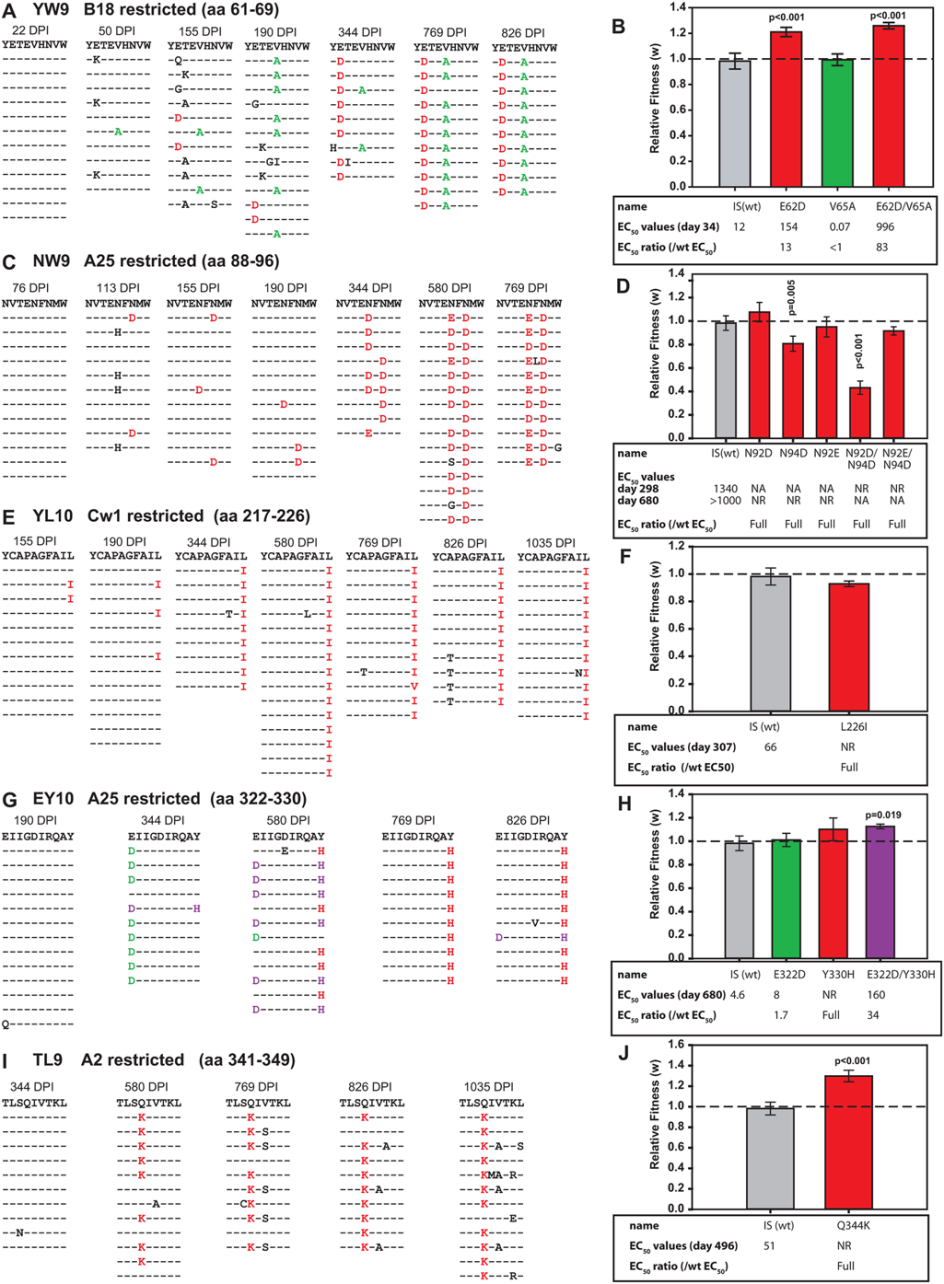
Ex vivo fitness, CTL escape phenotype and sequence evolution of *env* gp120 CTL escape mutants. The predicted amino acid sequences for 10–16 clones from different DPS from subject 1362 are presented for epitopes YW9 (A), NW9 (C), YL10 (E), EY10 (G), and TL9 (E). The effect of each mutation or combination of mutations on the CTL response to the mutant peptide was assessed by ELISpot (grey box in panels B, D, F, H, and J, respectively). ELISpot and EC_50_ concentrations were determined as described in the legend to Figure 4. Mutations that confer significant CTL escape are displayed in red text and bars in the respective panels. Mutations that did not confer significant escape are shown in green. Combinations of these mutations are shown in purple. Control experiments (gray bars) and significance testing were as described in the legend to Figure 4.

In stark contrast to the results for the p24 mutants, escape mutations in four (YW9, NW9, YL10, EY10, TL9) of five gp120 epitopes (Figure 7A, E, G, and I respectively) resulted in either no change in fitness or small, but statistically significant increases in fitness (Figure 7B, F, H, and J). Although escape mutations in the YW9, NW9, and YL10 epitopes appeared as early as 155 DPS in the virus population, escape mutations in EY10 and TL9 did not emerge until 580 DPS or around the same time frame as escape mutation in the Gag p24 epitopes, EW10 and QW11. In the case of YW9, two epitotic mutations evolved independently starting at 50 DPS (Figure 7A). The fitness neutral V65A virus may present a YW9 mutant peptide with enhanced CTL recognition based on ELISpot whereas E62D and E62D/V65A viruses, both with increased fitness, displayed peptides modestly (EC_50_ ratio = 13-fold) and were highly resistant (EC_50_ ratio>83 [13]) to CTL recognition, respectively (Figure 7B). Thus, the linkage of the E62D and V65A not only increased resistance to CTL pressure but also encoded for a more fit virus over V65A alone. However, apparent lack of CTL escape with V65A in YW9 (Figure 7A and B) as well as E322D in EY10 (Figure 7G and H; see below) suggests that these mutations may alter processing, MHC class I presentation, or may even emerge in response to another selective pressure such as humoral response. In particular, the E322D mutation within the EY10 epitope also maps to the base of the V3 loop and just adjacent to the crown of this loop (Figure 3A and B), i.e. a highly immunodominant region for antibody response. In contrast, V65A is found at the N-terminal end of a putative T-Helper/CD4+ epitope, H-2bxk or H-2sxd (Los Alamos HIV Molecular Immunology Database; www.hiv.lanl.gov/content/immunology). HLA class II alleles were not identified in this patient. Aside from these two mutations, CTL escape was conferred by the other seven mutations in CTL epitopes within gp120.

A similar pattern of epitotic mutations evolved in EY10 (Figure 7G and H). The first escape to appear was E322D (Figure 7G) with a minimal reduction in CTL recognition and without a fitness cost (Figure 7H). Subsequently (580 DPS), the C-terminal Y330H substitution appeared alone or linked to the N-terminal E322D (Figure 7G). Although the double mutant displayed increased fitness and conferred modest escape (Figure 7H), it was eventually overgrown by a fitness-neutral Y330H virus that appeared fully resistant to the EY10 CTL response.

Single CTL escape mutations appeared in YL10 and TL9 epitopes and dominated in all subsequent time points (Figure 7E and I, respectively). The L226I in YL10 did not significantly impact fitness (Figure 7F) whereas the Q344K in TL9 resulted in a 30% increase in replicative fitness (p<0.001) (Figure 7J).

In the fifth gp120 epitope, NW9, three persistent mutations were evaluated (Figure 7C), all of which conferred full escape (Figure 7D). The first to appear was N94D, which resulted a slight but significant decrease in fitness. Next to appear was N92D, which did not impact replicative fitness. The transient linkage of N92D and N94D resulted in the largest loss in fitness (Figure 7D). The N92E mutation, first detected at 344 DPS, replaced the N92D by 769 DPS in the virus population and then dominated with N94D (Figure 7C). This N92D/N94E combination restored viral fitness (Figure 7D). Thus, the virus appears to evolve into a form that escaped with poor fitness to one that retained immune escape but improved viral fitness. It is also interesting to note that the mutations at position 92 appeared to evolve sequentially - a single transition mutation was required for the N (AAT) to D (GAT) substitution, whereas a further transversion mutation was necessary to change D to E (GAG and GAA). This might explain why the N92D/N94D escape variant of low fitness emerged prior to the fitness neutral escape variant (N92E/N94D) (Figure 7C).

## Discussion

Evaluation of viral replicative fitness, commonly defined as relative viral replication success within cell culture, is becoming an important tool for the *ex vivo* study of host-virus interactions (reviewed by [46]). Several recent studies have shown or implied that CTL escape mutations can result in a loss of viral fitness [23]–[27], [31]–[34]. Our studies have improved upon this prior work by coupling longitudinal analysis of specific CTL escape/secondary mutations with their effects on replicative fitness. Instead of assessing the effects of single, discrete CTL escape mutations in a heterologous HIV-1 backbone, replicative fitness in this study was assessed with virus containing the autologous Gag p24 and Env gp120 sequences and the specific epitotic mutations as they appeared in the patient.

We found that the characteristics and impact of escape mutations differed between Gag p24 and Env gp120. Relative to those in p24, CTL escape mutations in gp120 appeared earlier (Figure 6), were more likely to give rise to full epitopic escape, and did not substantially diminish viral fitness. These findings uncover a dynamic evolutionary process of fitness-balanced CTL escape that is suggestive of several mechanistic pathways of viral adaptation to the host immune response.

First, the emergence rate of escape may be associated with the impact on viral fitness – the slower the appearance of escape, the greater the associated fitness cost. This is in agreement with previous studies that have shown that CTL escape mutations emerge more rapidly in less conserved genes of the virus [3]. For example, CTL escape mutations in HIV-1 Tat during asymptomatic disease had a neutral effect on Tat activity [47]. Differences in the appearance of CTL escape mutations have also been attributed to the processing of HIV-1 proteins found in early versus later stages of the retroviral life cycle [3],[6]. However, the latter hypothesis does not explain the early appearance of gp120 escape mutations, as gp120 is also a late protein in the retrovirus life cycle. Escape mutations did appear within the first 155 DPS within the Env YW9, NW9, YL10 and at least a year before the emergence of escape mutations in the Gag EW10 and QW11 epitopes. However, CTL escape was notably delayed in two other Env CTL epitopes. This delay in escape may have been associated with a weak CTL response to the wild type EY10 and TL9 epitopes (Figure 7).

Second, secondary mutations within and surrounding CTL epitopes tended to enhance viral fitness while maintaining or enhancing immunological escape. In the case of Env, the primary CTL escape mutations that appeared in 4 of 5 gp120 epitopes had a neutral or slightly positive impact on replication fitness. Subsequent mutations at the same or second sites within the epitope sometimes demonstrably enhanced escape and/or increased replication fitness (e.g., Y65A in YW9, Y330H in EY10). A similar pattern of eventual enhancement of viral fitness was even observed in the NW9 gp120 epitope where initial CTL escape mutations resulted in a slight fitness loss, but these amino acids were then replaced by those that enhanced fitness while maintaining escape. In the case of Gag, escape at the EW10 CTL epitope resulted in a significant loss in fitness. However, it was quickly compensated for by a V215L change C-terminal to the epitope. The latter change did not improve fitness, rather it appeared to restrict presentation of the epitope through a processing defect, allowing the epitopic escape mutation to revert back to the sensitive form. This hypothesis is supported by (1) prediction and direct evidence of increase cleavage within the EW10 epitope in the presence of V215L, (2) T cell responses (as measured by ELISpot) against EW10 in two HLA-A25 individuals with the epitope predicted to be in a “presentable” configuration, i.e., with valine at position 215, and by (3) the lack of T cell responses against EW10 in another two HLA-A25 individuals with leucine at this position, the putative configuration that disrupts processing [31]. It should be noted leucine is a more common amino acid at position 215 in the HIV-1 sequence database (89% L, 10% V), suggesting that this epitope is unfortunately unavailable to the immune recognition in most HIV-1 infected individuals. Although an EW10 processing defect is likely with V215L, T cell recognition of target cells synthesizing intact and mutant epitopes will be necessary to firmly establish this point.

Third, CTL escape mutations and secondary mutations in the EW10 and QW11 sites emerged in regions of the p24 protein accommodating some genetic change as opposed to more functionally conserved sites. In general, p24 mutations in N terminal β hairpin/helix 1 (site of CTL epitope, QW11) and in helix 4 (site of EW10) [36] (Figure 2B) do not alter virus core morphology or assembly based on a study by von Schwedler et al. 2003 [41]. In contrast, gross disruptions in virus core structure and drastic decreases in infectivity were associated with mutations in helix 2 (i.e. site of CTL epitope VL8) and helix 8 (site of FK10) (Figure 2B) [41]. This study may provide preliminary evidence as to why escape variants do not emerge in the VL8 and FK10 epitopes of PIC1362 despite strong CTL recognition for at least three years of infection [6]. Further structural/functional analyses are required to define the impact of these mutations on specific perturbations in p24 structure and defects in various p24 functions during virus replication. Finally, in support of this structural constraint for CTL escape in Gag, recent studies on SHIV evolution in macaque infections suggest that evolutionary pathways to CTL escape is more restricted with gag than with env [48].

Recent findings have correlated lower viral loads with CTL responses directed against the more conserved Gag rather than those directed against the more heterogeneous Env [49]. Furthermore, responses against subdominant viral epitopes are more closely related to virologic control than immunodominant epitopes [50]. However, our recent results have shown that Env is a CTL target that has been under-appreciated due to the lack of consensus peptide sets that accommodate its very high level of diversity [13]. Furthermore, there are obviously overlaps between MHC class I and II epitopes in *env* which may influence *env* versus *gag* evolution. The current study reconciles these observations through demonstration that CTL responses against Env, however immunodominant early in infection, may have little impact on virus fitness and thus contribute little to virologic control (Figure S4 online). Furthermore, any viral load decreases due to CTL pressure directed against gp120 may be quickly eroded due to rapid epitopic escape and emergence of compensatory mutations. Thus, sustained virological control exerted by CTL is likely due to continued immune pressure on the more structurally constrained viral proteins, e.g., in the Gag p24 protein. Ultimately, maintenance of HIV-specific CTL pressure may be indirectly governed by replicative fitness. Continued virologic suppression during asymptomatic infection and slow emergence of epitopic escape mutations in Gag is likely due to the consequence of their high fitness costs.

## Materials and Methods

### Study subjects and specimens

Study subjects PIC1362, PIC1349, PIC1052 and PIC1483 were participants in a natural history study at the University of Washington Primary Infection Clinic (PIC). HLA alleles were determined by allele-specific PCR to be A*0201, A*2501, B*1801, B*5101, Cw*0102, Cw*1203 for PIC1362 [6]; A*02, A*25, B*58, B*62 (likely 1501), Cw*04, Cw*07 for PIC1349; A*25, A*30, B*07, B*35, Cw*04, Cw*07 for PIC1052; and A*0101, A*2501, B*3801, B*3901, Cw*1203, Cw*1203 for PIC1483. Virologic, immunologic, and other clinical parameters of PIC1362 have been reported previously [6],[13]. For the current study, viral DNA was obtained from PIC1362 PBMC at 34 and 298 days post onset of acute symptoms of primary HIV infection (DPS). This study was approved by the Institutional Review Boards of the University of Washington, Fred Hutchinson Cancer Research Center, and Case Western Reserve University, and all subjects provided informed consent for participation in the study.

### Construction and propagation of chimeric HIV-1

The gp120-encoding region of *env* (nucleotides 6347–7802, HIV-1_HXB2_) and the p24-encoding region of *gag* (nucleotides 1089–2022, HIV-1_HXB2_) of the initial/infecting strain (IS) were PCR amplified from PBMC DNA of PIC1362 at 34 and 298 DPS, respectively. PCR reactions were conducted using Platinum® Taq DNA Polymerase High Fidelity following the manufacturer's recommendation (Invitrogen). Primers *ENV* START and ED12-EXT [38], and GAD5 and GAD4 (Table S1 online) were used for the gp120 and p24 amplifications, respectively.

PCR products were inserted into two versions of the NL4-3 HIV-1 molecular clone, pNL4-3_vifA_ and pNL4-3_vifB_, differing at only six synonymous nucleotides in the HIV-1 *vif* gene, using a previously described yeast-based recombination system [38] (Figure S1 online).

Infectious HIV-1 chimeras were produced by transfection of NL4-3/gp120 and NL4-3/p24 into 293T cells as previously described [38]. NL4-3/p24 viruses were briefly propagated (≤10 days) on U87.CD4.CXCR4 cells. NL4-3/gp120 viruses were briefly propagated on U87.CD4.CCR5 cells since each PIC1362 *env* gene examined from this subject had been found to be genotypically R5-tropic [13]. Infections were monitored by a reverse transcriptase (RT) activity assay and supernatant was collected at peak RT activity (≤10 days) and frozen at −140°C. Infectious virus TCID_50_ [51] were determined on phytohemagglutinin (PHA) and interleukin-2 (IL-2) stimulated PBMC as previously described [52].

In order to test for genetic stability during replication, each virus was used to infect 100,000 PHA and IL-2 stimulated PBMC [52] in triplicate at an MOI of 0.005. After 10 days, DNA was extracted from infected cells and the p24 and gp120 regions of proviral DNA were PCR amplified as previously described [52], with primers Gseq1 and Gseq2 for p24 and primers Eseq1 and Eseq2 for gp120. Sequence analysis on these PCR products confirmed that in all cases, the proviral sequences were identical to that of the infecting virus, i.e., none of the CTL escape mutations in NL4-3/gp120 or NL4-3/p24 viruses reverted to the pre-escape amino acids during the 10 day infection.

### HIV fitness and competition assays

Dual infection and competition assays were conducted as previously described [52],[53]. Dual virus detection by vif synonymous substitutions has been recently described [39],[40]. Details of this competition and dual virus detection as pertaining to this study are provided in Figure S2 online.

### HIV-1 population sequencing

Detailed clonal nucleotide sequencing of PIC1362 HIV-1 populations was described previously by Liu *et al.* [13]. Population nucleotide sequencing of HIV-1 Gag p24 was performed for this study on plasma and PBMC of PIC1349, PIC1052 and PIC1483. Briefly, HIV-1 RNA was extracted from plasma using the QIAamp Viral RNA Mini Kit (Qiagen) and PBMC DNA containing HIV-1 provirus was extracted using the QIAamp DNA Blood Mini Kit (Qiagen). PCR and RT-PCR were conducted as previously described [52] with first external primers RTG1 and RTG2 followed by internal primers RTG3 and RTG4 (Table S1). Nucleotide sequencing was performed by Davis Sequencing, Inc. using an ABI 3700 DNA sequencer and sequence chromatograms were examined using BioEdit v. 5 (Tom Hall, North Carolina State University).

### Peptide processing ex vivo and in silico

Peptides of 27 and 40 amino acids in length and representing positions 200 to 226 (or positions 195 to 234) in the Env gp120 sequence were used in silico in the NetChop 3.0 C-term peptide processing algorithm (http://www.cbs.dtu.dk/services/NetChop/). C-term 3.0 network is trained with a database consisting of 1260 publicly available MHC class I ligands (using only C-terminal cleavage site of the ligands).

Peptides (27 mer) derived from the wild type sequence of PIC1362 or harboring the E207D/V215L or V215L/I223V mutations were synthesized by Bio-Synthesis Inc, (Lewisville, TX). Purity (>95%) and peptide sequences were confirmed by mass spectrometry analysis. Cytosol from PBMC of healthy donors was prepared in detergent-free buffer by breaking cells with glass beads and purified by mutiple centrifugations as described previously [45]. Protein concentration and content as well as antigen processing activities were checked by Western blot and enzymatic assays as described previously [45]. 8 nmol of purified peptides were degraded with 40 µg cytosol at 37°C for up to 60 minutes. The peptides present in the digestion mix at different time points were identified and quantified by reverse-phase high pressure liquid chromatography (RP-HPLC) and mass spectrometry (Taplin Mass Spectrometry Facility, Harvard Medical School) [45].

### Online supplemental material

A detailed graphical description of the methods for clonal sequence analyses, putative CTL escape peptide testing, production of infectious chimeric viruses and introduction of escape mutations is provided in Figure S1 online. Details of virus competitions and dual virus detection conducted in this study are provided in Figure S2 online. Nucleotide sequences of primers used in this study are provided in Table S1 online.

## Supporting Information

Figure S1 Detailed strategy for analyzing patient 1362 CTL escape, sequence evolution and ex vivo HIV-1 fitness. Steps 1 to 5 have previously been reported by Liu *et al.* [13].
(1.11 MB PDF)

Figure S2 Competitive ex vivo HIV-1 fitness assay. Initial/infecting strain (IS) and mutant chimeric viruses in the vif B and vif A backgrounds, respectively, were competed in dual infections and replicated as monoinfections at an MOI of 0.005 (A). The resulting proportions of IS vif B and mutant vif A were determined by heteroduplex tracking assay (HTA) targeting the vif gene (B). Proviral DNA was amplified by nested PCR and these products were annealed to a ^32^P radiolabeled probe complementary to either the vif A or vif B sequence. Differences in the vif sequence at the 5′ end of the probe cause the heteroduplex (probe vif B annealed to vif A DNA) to migrate more slowly in a polyacrylamide gel, compared to the homoduplex (probe vif B annealed to vif B DNA). HTA results for competitions of gp120-IS (vif B) against gp120 NW9 epitope mutants (vif A) are displayed using the vif B probe (C). Relative fitness (w) was then calculated from the intensity of the virus-specific bands in competition in relation to the intensity of the monoinfection bands as described here (D) and previously [36],[53]. Relative fitness values for the mutant viruses were then plotted such that w>1 indicates greater fitness of the mutant while w<1 indicates greater fitness of the IS (E). The gray bar indicates the control in which gp120-IS in the vif A and vif B backgrounds were competed against each other, resulting in a nearly equal relative fitness (w = 1).
(2.94 MB PDF)

Figure S3 Evolution of the EW10 peptide and ELISpot response in PIC1362. (A) The relative proportions of the E207D and V215L in the HIV-1 population of PIC1362 has been previously published but presented in this panel to compare with (B) the ELISpot responses to 2 µg/ml of the EW10 peptide in PBMCs derived from 496, 713, 826, 1329, and 1501 DPS.
(0.33 MB PDF)

Figure S4 Timing of the first detected appearance of CTL escape mutations in p24 and gp120 relative to patient viral load and CD4 cell count. Plasma viral load and CD4 cell count are shown relative to the days post acute symptoms (A). On the same scale, relative *ex vivo* fitness of each p24 and gp120 CTL escape mutation observed in patient 1362 is shown at the timepoint of its first detection by clonal sequencing (B).
(0.61 MB PDF)

Table S1 Primers used in this study.
(0.04 MB DOC)

Protocol S1 Supplementary methods.
(0.04 MB DOC)
