# Supplementary material for: Variable Fitness Impact of HIV-1 Escape Mutations to Cytotoxic T Lymphocyte (CTL) Response
Source: PLoS Pathog. 2009 Apr 3;5(4):e1000365. doi: 10.1371/journal.ppat.1000365 (PMC2659432; doi:10.1371/journal.ppat.1000365)
Supplement: Figure S1 — Detailed strategy for analyzing patient 1362 CTL escape, sequence evolution and ex vivo HIV-1 fitness. Steps 1 to 5 have previously been reported by Liu et al. [13]. (1.11 MB PDF) [file ppat.1000365.s001.pdf]

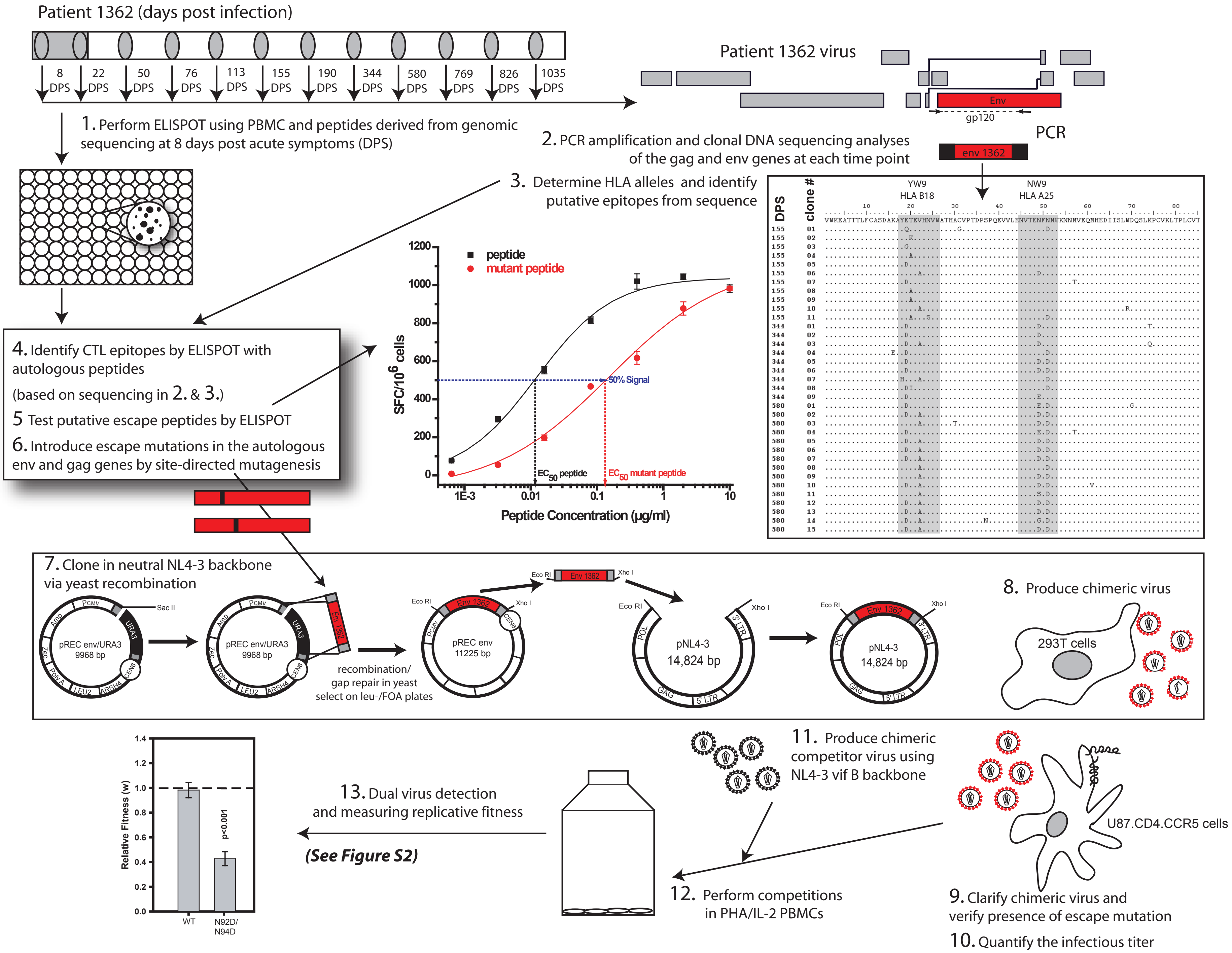

Figure S1. Strategy for analyzing patient 1362 CTL escape, sequence evolution and ex vivo HIV-1 fitness. Steps 1 to 5 have previously been reported by Liu et al.
