# Supplementary material for: Variable Fitness Impact of HIV-1 Escape Mutations to Cytotoxic T Lymphocyte (CTL) Response
Source: PLoS Pathog. 2009 Apr 3;5(4):e1000365. doi: 10.1371/journal.ppat.1000365 (PMC2659432; doi:10.1371/journal.ppat.1000365)
Supplement: Figure S2 — Competitive ex vivo HIV-1 fitness assay. Initial/infecting strain (IS) and mutant chimeric viruses in the vif B and vif A backgrounds, respectively, were competed in dual infections and replicated as monoinfections at an MOI of 0.005 (A). The resulting proportions of IS vif B and mutant vif A were determined by heteroduplex tracking assay (HTA) targeting the vif gene (B). Proviral DNA was amplified by nested PCR and these products were annealed to a 32P radiolabeled probe complementary to either the vif A or vif B sequence. Differences in the vif sequence at the 5′ end of the probe cause the heteroduplex (probe vif B annealed to vif A DNA) to migrate more slowly in a polyacrylamide gel, compared to the homoduplex (probe vif B annealed to vif B DNA). HTA results for competitions of gp120-IS (vif B) against gp120 NW9 epitope mutants (vif A) are displayed using the vif B probe (C). Relative fitness (w) was then calculated from the intensity of the virus-specific bands in competition in relation to the intensity of the monoinfection bands as described here (D) and previously [36],[53]. Relative fitness values for the mutant viruses were then plotted such that w>1 indicates greater fitness of the mutant while w<1 indicates greater fitness of the IS (E). The gray bar indicates the control in which gp120-IS in the vif A and vif B backgrounds were competed against each other, resulting in a nearly equal relative fitness (w = 1). (2.94 MB PDF) [file ppat.1000365.s002.pdf]

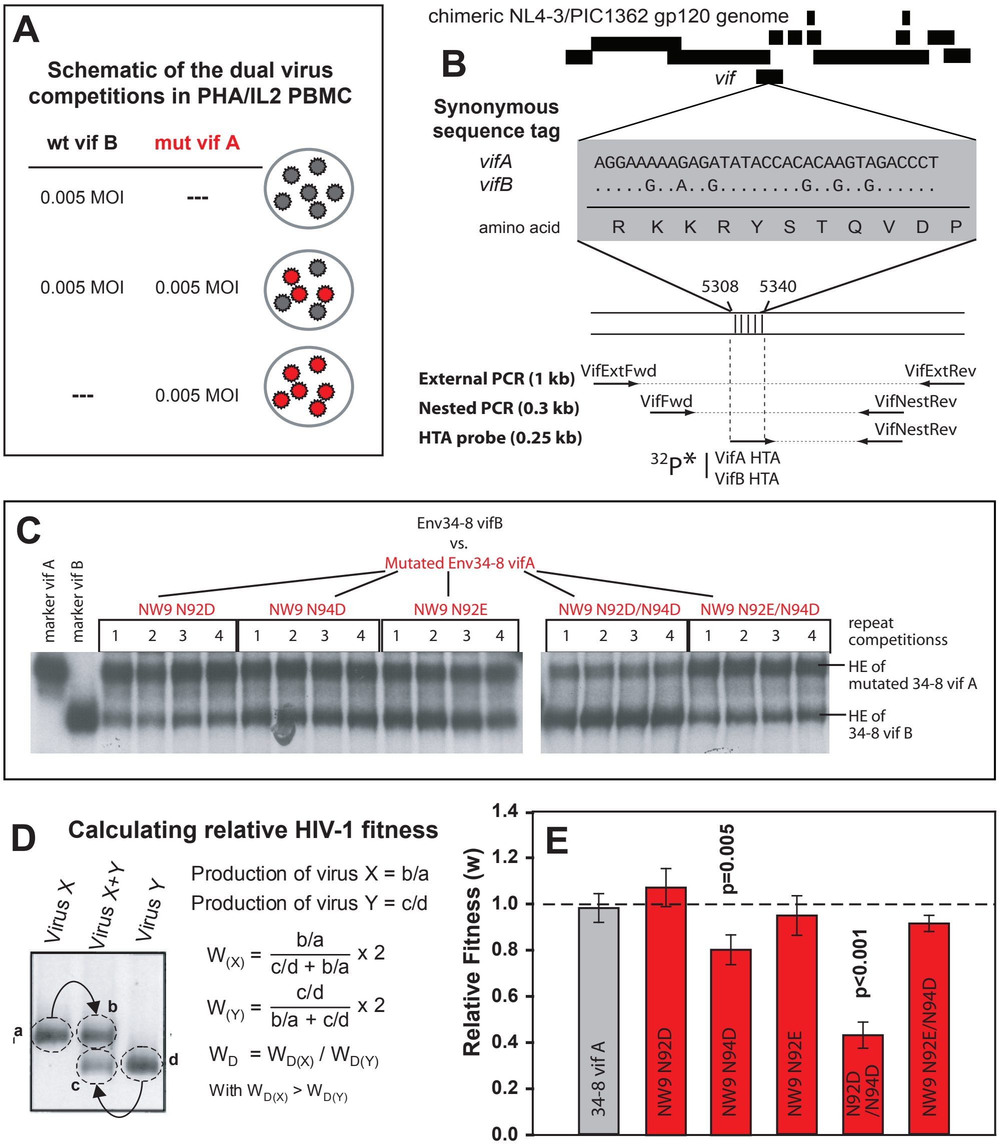

$$W_{(X)} = \frac{b/a}{c/d + b/a} \times 2$$

$$W_{(Y)} = \frac{c/d}{b/a + c/d} \times 2$$

$$W_D = W_{D(X)} / W_{D(Y)}$$

Figure S2. Competitive ex vivo HIV-1 fitness assay. Initial/infecting strain (IS) and mutant chimeric viruses in the vif B and vif A backgrounds, respectively, were competed in dual infections and replicated as monoinfections at an MOI of 0.005 (A). The resulting proportions of IS vif B and mutant vif A were determined by heteroduplex tracking assay (HTA) targetting the vif gene (B). Proviral DNA was amplified by nested PCR and these products were annealed to a P32 radiolabelled probe complementary to either the vif A or vif B sequence. Differences in the vif sequence at the 5' end of the probe cause the heteroduplex (probe vif B annealed to vif A DNA) to migrate more slowly in a polyacrylamide gel, compared to the homoduplex (probe vif B annealed to vif B DNA). HTA results for competitions of gp120-IS (vif B) against gp120 NW9 epitope mutants (vif A) are displayed using the vif B probe (C). Relative fitness (w) was then calculated from the intensity of the virus-specific bands in competition in relation to the intensity of the monoinfection bands as described here (D) and previously105, 106. Relative fitness values for the mutant viruses were then plotted such that w>1 indicates greater fitness of the mutant while w<1 indicates greater fitness of the IS (E). The gray bar indicates the control in which gp120-IS in the vif A and vif B backgrounds were competed against each other, resulting in a nearly equal relative fitness (w = 1).
