# Supplementary material for: Variable Fitness Impact of HIV-1 Escape Mutations to Cytotoxic T Lymphocyte (CTL) Response
Source: PLoS Pathog. 2009 Apr 3;5(4):e1000365. doi: 10.1371/journal.ppat.1000365 (PMC2659432; doi:10.1371/journal.ppat.1000365)
Supplement: Figure S3 — Evolution of the EW10 peptide and ELISpot response in PIC1362. (A) The relative proportions of the E207D and V215L in the HIV-1 population of PIC1362 has been previously published but presented in this panel to compare with (B) the ELISpot responses to 2 µg/ml of the EW10 peptide in PBMCs derived from 496, 713, 826, 1329, and 1501 DPS. (0.33 MB PDF) [file ppat.1000365.s003.pdf]

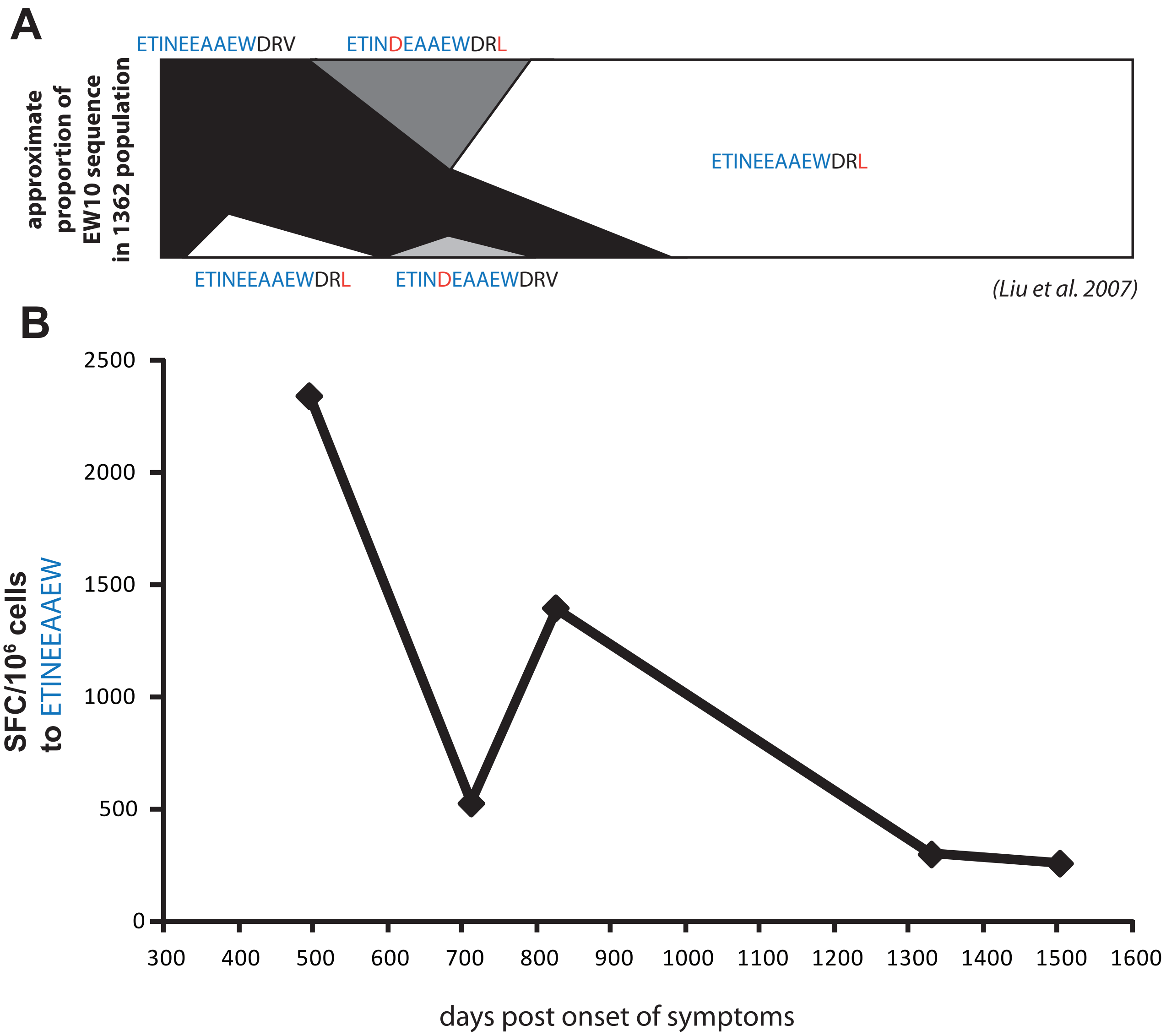


**Figure S3.** Evolution of the EW10 peptide and ELISpot response in PIC1362. (A) The relative proportions of the E207D and V215L in the HIV-1 population of PIC1362 has been previously published but presented in this panel to compare with (B) the ELISpot responses to 2 μg/ml of the EW10 peptide in PBMCs derived from 496, 713, 826, 1329, and 1501 DPS.
