# Supplementary material for: Variable Fitness Impact of HIV-1 Escape Mutations to Cytotoxic T Lymphocyte (CTL) Response
Source: PLoS Pathog. 2009 Apr 3;5(4):e1000365. doi: 10.1371/journal.ppat.1000365 (PMC2659432; doi:10.1371/journal.ppat.1000365)
Supplement: Figure S4 — Timing of the first detected appearance of CTL escape mutations in p24 and gp120 relative to patient viral load and CD4 cell count. Plasma viral load and CD4 cell count are shown relative to the days post acute symptoms (A). On the same scale, relative ex vivo fitness of each p24 and gp120 CTL escape mutation observed in patient 1362 is shown at the timepoint of its first detection by clonal sequencing (B). (0.61 MB PDF) [file ppat.1000365.s004.pdf]

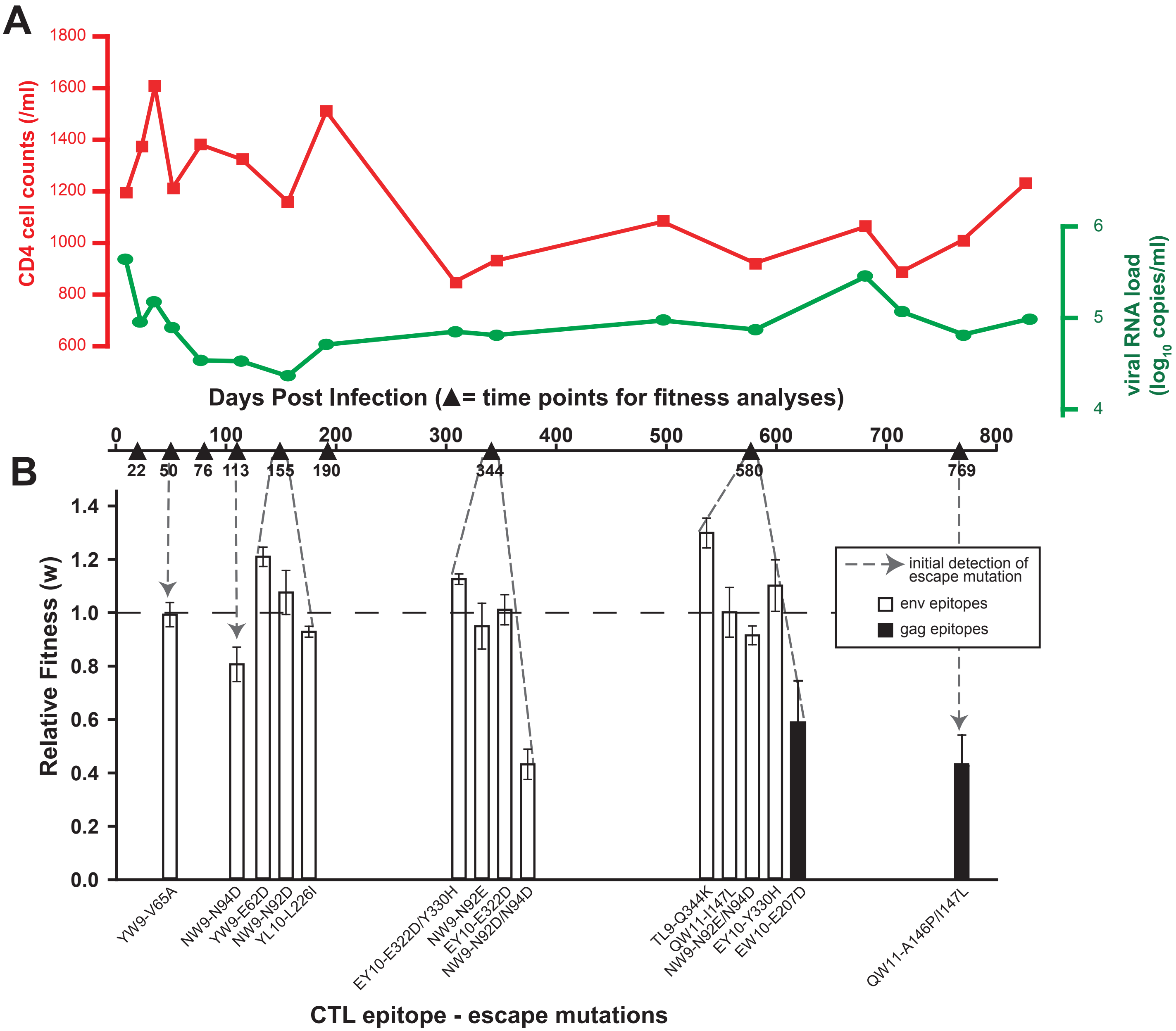

Figure S4. Timing of the first detected appearance of CTL escape mutations in p24 and gp120 relative to patient viral load and CD4 cell count. Plasma viral load and CD4 cell count are shown relative to the days post acute symptoms (A). On the same scale, relative ex vivo fitness of each p24 and gp120 CTL escape mutation observed in patient 1362 is shown at the timepoint of its first detection by clonal sequencing (B).
