# Supplementary material for: Variable Fitness Impact of HIV-1 Escape Mutations to Cytotoxic T Lymphocyte (CTL) Response
Source: PLoS Pathog. 2009 Apr 3;5(4):e1000365. doi: 10.1371/journal.ppat.1000365 (PMC2659432; doi:10.1371/journal.ppat.1000365)
Supplement: Table S1 — Primers used in this study. (0.04 MB DOC) [file ppat.1000365.s005.doc]

**Table S1.** Primers used in this study.

| Primer name | Gene | Orientation | Nucleotide Sequence | 5’ end position† | 3’ end position† |
| --- | --- | --- | --- | --- | --- |
| GAD5 | gag | forward | GTAAAAGACACCAAGGAAGC | 1064 | 1088 |
| GAD4 | gag | reverse | CCACATTTCCAACAGCC | 2039 | 2023 |
| Gseq1 | gag | forward | CAATAGCAGTCCTCTATTGTGTGC | 1031 | 1054 |
| Gseq2 | gag | reverse | TAGCCTGTCTCTCAGTACAATCTTTC | 2083 | 2058 |
| RTG1 | gag | forward | GTATGGGCAAGCAGGGAGC | 892 | 910 |
| RTG2 | gag | reverse | GGGTCGTTGCCAAAGAGTG | 2279 | 2261 |
| RTG3 | gag | forward | TAGAGGTAAAAGACACCAAGGAAGC | 1064 | 1088 |
| RTG4 | gag | reverse | CCTTTCCACATTTCCAACAGCC | 2044 | 2023 |
| Eseq1 | env | forward | CATGCTCCTTGGGATGTTGATG | 6281 | 6302 |
| Eseq2 | env | reverse | TTCTGCTGCTGCACTATACCAGAC | 7882 | 7859 |
| VifExtFwd | vif | forward | GCAAAGCTCCTCTGGAAAGGTGAAGGG | 4944 | 4970 |
| VifExtRev | vif | reverse | CTTCCACTCCTGCCCAAGTATCCC | 5733 | 5710 |
| VifFwd | vif | forward | GAAAGAGACTGGCATTTGGGTCAGGG | 5266 | 5291 |
| VifNestRev | vif | reverse | GTCTTCTGGGGCTTGTTCCATCTGTCC | 5579 | 5553 |
| VifA-HTA | vif | forward | AAAGAGATATACCACACAAGTAGACCCTGACC | 5313 | 5344 |
| VifB-HTA | vif | forward | GAAAAGGTATACCACGCAGGTGGACCC | 5313 | 5339 |

† Numbering corresponds to positions in HIV-1 reference strain HXB-2 full length genome.
