# Supplementary material for: Variable Fitness Impact of HIV-1 Escape Mutations to Cytotoxic T Lymphocyte (CTL) Response
Source: PLoS Pathog. 2009 Apr 3;5(4):e1000365. doi: 10.1371/journal.ppat.1000365 (PMC2659432; doi:10.1371/journal.ppat.1000365)
Supplement: Protocol S1 — Supplementary methods. (0.04 MB DOC) [file ppat.1000365.s006.doc]

**Protocol S1: Supplementary Methods**

HIV fitness and competition assays

Dual infection and competition assays were conducted as previously described ^1, 2^. Briefly, each chimeric NL4-3_vifA_/p24 virus containing the CTL escape mutation was competed against NL4-3_vifB_/p24-IS, while NL4-3_vifA_/gp120 viruses containing CTL escape mutations were competed against NL4-3_vifB_/gp120-IS reference strains containing the baseline viral sequence from subject 1362. Dual infections were conducted in quadruplicate at a 1:1 ratio, and each virus was added at a multiplicity of infection (MOI) of 0.005 to 100,000 PBMC treated with PHA (2 ug/ml) and IL-2 (1 ng/ml) (Figure 2A). Monoinfections of each virus were also performed at an MOI of 0.005. An RT activity assay was performed on supernatant from each dual infection to monitor total virus production. Cells and supernatant were harvested and stored at -80°C on day 10.

A sensitive and quantitative heteroduplex tracking assay (HTA) was used to differentially quantify virus production of NL4-3_vifA_ and NL4-3_vifB_ viruses in dual-infections (Supplementary Figure 2). Briefly, the *vif* region of the chimeric viruses was amplified by nested PCR from the PBMC DNA of all dual and monoinfections using conserved primers. External primers were VifExtFwd and VifExtRev and the internal primers were VifFwd and VifNestRev. PCR conditions have been described previously ^1^. Radiolabelled [^32^P] vif DNA probes were produced by PCR, using γ-[^32^P] ATP end labeled sense primers VifA-HTA or VifB-HTA, and a cold antisense primer, VifNestRev, as previously described ^1^. PCR products from all dual and monoinfections were then mixed with either vifA or vifB radiolabeled probe, denatured, annealed and separated by electrophoresis on a nondenaturing 6% polyacrylamide gel. Sequence differences in the probe and target DNA cause heteroduplexes to migrate more slowly than homoduplexes, thus allowing separation and quantification of virus-specific bands. Bands specific to NL4-3_vifA_ and NL4-3_vifB_ viruses were quantified using a Molecular Imager FX (Bio-Rad) phosphorimager. To normalize probe hybridization, virus production was compared between dual infection and monoinfections. Relative fitness (w) was calculated by dividing the production of an individual virus (b/a or c/d, Figure S2D online) in a dual infection by the total virus production (b/a + c/d, Figure S2d online) and then divided by the initial proportion in the inoculum (0.5).

Construction of chimeric HIV-1

Briefly, gp120 or p24 PCR products were co-transfected into yeast with plasmid pRECenv/URA3 linearized with Sac II or pRECgag/URA3 linearized with Bpu1102I, respectively. Recombination directed by the yeast gap repair mechanism resulted in the replacement of URA3 with gp120 or p24, and yeast colonies with this replacement were selected on leu-/FOA plates. Twenty-four cloned gp120 or p24 genes in pREC were sequenced using primers Eseq1 and Eseq2 for gp120 and primers Gseq1 and Gseq2 for p24 (primer sequences provided in Supplementary Table 1). Clones pRECenv34-8 and pRECgag298-3 had the exact gp120 or p24 sequence of the major viral sequences of PIC1362 at 8 DPS ^3^. Therefore, these clones were chosen as representative of the initial/infecting strain (IS) for PIC1362. A cassette containing the HIV gene segment and flanking sequences of pRECenv34-8 and pRECgag298-3 were then removed by restriction enzyme digestion with Eco RI and Xho I, or BssHII and SbfI, respectively, and ligated into pNL4-3_vifA_ and pNL4-3_vifB_ (Figure S1 online). Point mutations conferring CTL escape were introduced into the gp120 and p24 sequences in pRECenv and pRECgag by PCR mutagenesis and then inserted into pNL4-3_vifA_ as described above.

Reference List

1. Quinones-Mateu,M.E. *et al.* A dual infection/competition assay shows a correlation between ex vivo human immunodeficiency virus type 1 fitness and disease progression. *J. Virol.* **74**, 9222-9233 (2000).

2. Troyer,R.M. *et al.* Changes in human immunodeficiency virus type 1 fitness and genetic diversity during disease progression. *J. Virol.* **79**, 9006-9018 (2005).

3. Liu,Y. *et al.* Selection on the human immunodeficiency virus type 1 proteome following primary infection. *J. Virol.* **80**, 9519-9529 (2006).
